# Wavefront correction with image-based interferometric focus sensing in two-photon microscopy

**DOI:** 10.1515/nanoph-2024-0738

**Published:** 2025-03-11

**Authors:** Ruiwen Yang, Yanlong Yang, Tengfei Wu, Yang Zhang, Dan Dan, Junwei Min, Xianghua Yu, Taiqiang Dai, Liang Kong, Li Li, Baoli Yao

**Affiliations:** State Key Laboratory of Ultrafast Optical Science and Technology, Xi’an Institute of Optics and Precision Mechanics, Chinese Academy of Sciences, Xi’an 710119, China; University of Chinese Academy of Sciences, Beijing 100049, China; Department of Oral and Maxillofacial Surgery, State Key Laboratory of Oral & Maxillofacial Reconstruction and Regeneration, National Clinical Research Center for Oral Diseases, Shaanxi Clinical Research Center for Oral Diseases, School of Stomatology, The Fourth Military Medical University, Xi’an 710032, China; Department of Ophthalmology, The First Affiliated Hospital of Xi’an Jiaotong University, Xi’an 710061, China

**Keywords:** two-photon microscopy, adaptive optics, interferometric focus sensing, image-based metric

## Abstract

Adaptive optics is a technology that corrects wavefront distortions to enhance image quality. Interferometric focus sensing (IFS), a relatively recently proposed method within the field of adaptive optics, has demonstrated effectiveness in correcting complex aberrations in deep tissue imaging. This approach determines the correction pattern based on a single location within the sample. In this paper, we propose an image-based interferometric focus sensing (IBIFS) method in a conjugate adaptive optics configuration that progressively estimates and corrects the wavefront over the entire field of view by monitoring the feedback of image quality metrics. The sample conjugate configuration allows for the correction of multiple points across the full field of view by sequentially measuring the correction pattern for each point. We experimentally demonstrate our method on both the fluorescent beads and the mouse brain slices using a custom-built two-photon microscope. We show that our approach has a large effective field of view as well as more stable optimization results compared to the region of interest based method.

## 1 Introduction

Due to the inhomogeneous distribution of the refractive index in a biological sample, light is scattered during its propagation, resulting in a significant degradation of image quality and a limited imaging depth. In recent years, several methods have been proposed to address this issue [1], [2], [3]. One strategy is to use a longer wavelength excitation, which suffers less from scattering and can be used to increase the imaging depth, such as two-photon microscopy (TPM) [4], [5]. Another is to utilize adaptive optics (AO) [6], which, having been successfully used to correct for atmospheric turbulence in astronomy, has also been introduced to correct the aberrations in biological imaging [7], [8], [9], [10], [11]. Recent studies have shown that combining TPM with sensorless AO can significantly enhance the performance of deep tissue imaging.

AO can be categorized into two main strategies, i.e., the direct method and the indirect method [9], [12]. The direct method directly measures wavefront aberrations using a wavefront sensor, followed by compensation with a wavefront modulator. This approach offers a rapid response speed but requires the use of a wavefront sensor, which introduces additional complexity to the system. Conversely, the indirect method, often called sensorless AO, estimates wavefront aberrations through an iterative process. This involves loading a series of phase patterns onto the wavefront modulator while monitoring the signal with specific metrics such as peak intensity or image sharpness. Employing image quality as a metric [13], [14] represents a result-oriented strategy, enabling wavefront correction across the entire field of view (FoV) simultaneously with modal decomposition in the pupil plane. Despite a slower response than the direct method, the sensorless AO strategy offers a simpler hardware setup and is applicable to opaque tissues.

A classic solution for sensorless AO is to use the mean image intensity as the metric and the Zernike modes as the basis for the aberration representation. The best correction can be achieved by measuring the maximum metrics over different modes, typically fitted to quadratic functions. This approach has been applied in two-photon microscopy [13], structured illumination microscopy [15], [16], and third harmonic generation microscopy [17]. This method performs best when the aberration amplitude is small [18]. When imaging deeper into tissue, new correction methods are required to measure and correct the complex aberrations. One such method is known as interferometric focus sensing (IFS) [19], [20]. The principle of IFS involves splitting the excitation light into two beams that interfere at the focal plane, with one beam being scanned against the other using a tip/tilt mirror. The complex-valued electric field at the focal plane can be determined by multiple intensity measurements at different phase delays between the two beams using an electro-optic modulator. An alternative simplified setup, known as dynamic adaptive scattering compensation holography (DASH) [21], [22], was proposed by exploiting a liquid crystal spatial light modulator (SLM) for relative beam scanning and phase delaying. This approach updates the phase pattern after each mode, but the overall performance is limited by the slow framerate of the SLM. The IFS system updates its correction phase based on a specific metric derived from the feedback signal from a small region of interest (ROI) in the sample. The corrected image is acquired by rescanning the same region until the aberration is compensated.

In general, image-based AO is effective for correcting low-order aberrations with a single wavefront modulator at the pupil-conjugate plane. However, due to the optical memory effect [23], wavefront correction is optimal within the region known as the isoplanatic patch. For spatially varying aberrations, conjugate AO has been developed to provide a large FoV correction by placing a wavefront modulator conjugated to the main source of aberrations [24], [25]. The approach has been applied to widefield or scanning microscopy with various reflective modulators, such as deformable mirrors [26], [27] or liquid crystal SLMs [28]. With transmissive modulators, such as adaptive lenses [29] or deformable phase plates, compact conjugate AO has been demonstrated as an extension to a commercial microscope [30]. A similar approach has been implemented in DASH with six selected points for correction, and the optimization was performed sequentially [31].

In this paper, we propose an image-based interferometric focus sensing (IBIFS) method for TPM, which combines the advantages of DASH for point spread function (PSF) tuning and image-based metrics as feedback signals for optimization. IBIFS utilizes image metrics across the entire FoV to correct the aberrations progressively during the imaging process. Additionally, we employ a conjugate AO scheme, in which the SLM is conjugated to a plane between the objective and the focal plane in our setup. In this manner, the overall optimization trades off better correction within a relatively small ROI against the average improvement of image quality over the full FoV. We demonstrate the IBIFS approach using a custom-built two-photon microscope with a resonant-galvo scanner and show its effectiveness in correcting aberrations in fluorescent beads and mouse brain slices. The IBIFS method may be beneficial to deep tissue imaging.

## 2 Methods

**Figure 1:**
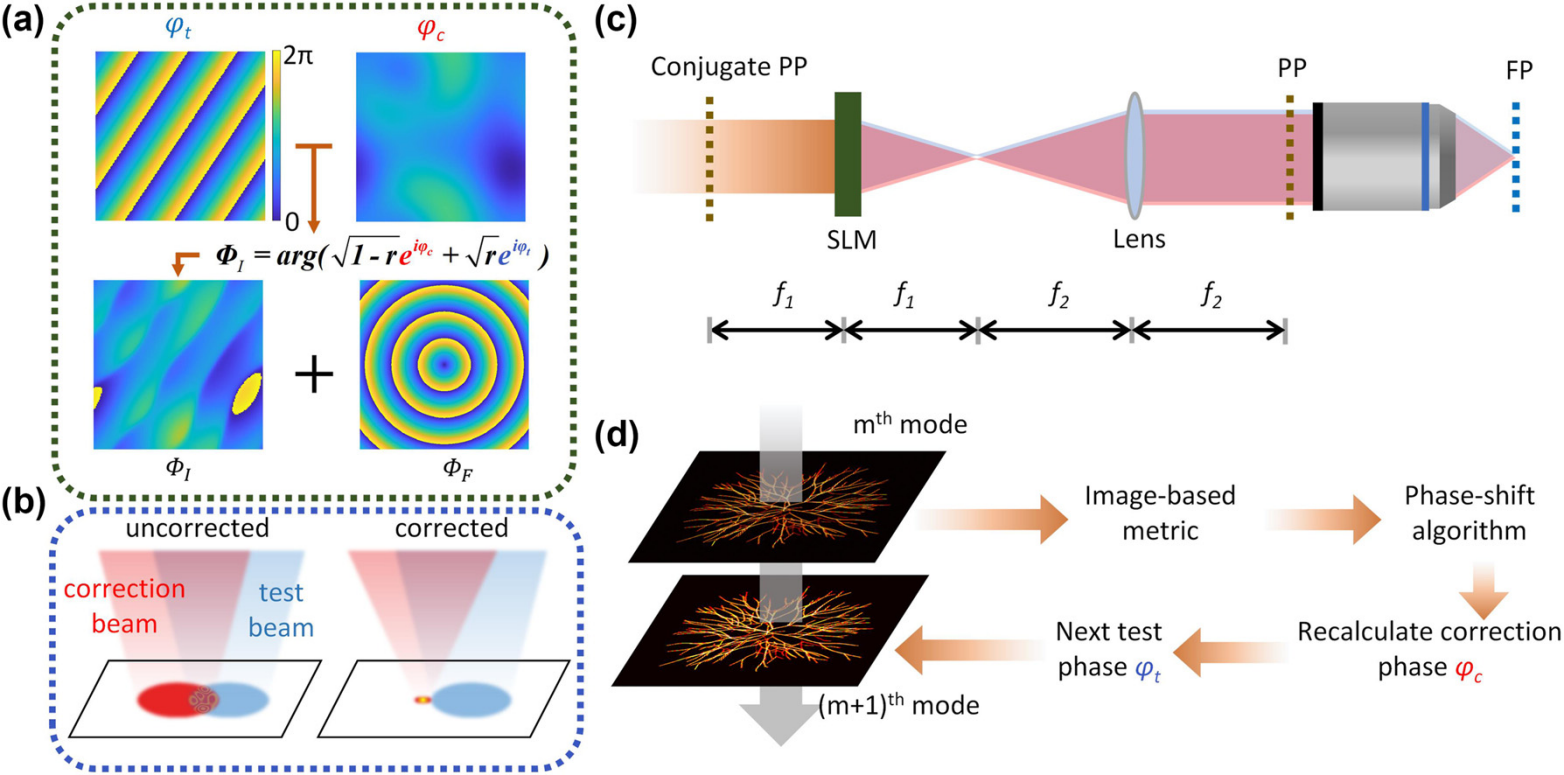
Principle of the IBIFS method. (a) Schematic of the phases addressed on the SLM. *φ*_*c*_, correction phase; *φ*_*t*_, test phase; Φ_*I*_, computer-generated hologram phase derived from the correction phase and test phase; Φ_*F*_, Fresnel lens phase. The SLM is loaded with a superimposed phase of Φ_*I*_ and Φ_*F*_. (b) The excitation light is separated into the correction beam (red) and the test beam (blue). Two beams interfere on the focal plane, and the correction beam iteratively converges with a focal point. (c) Schematic of optical layout, PP, pupil plane; FP, focal plane, *f*_1_, the focal length of Fresnel lens phase on SLM, *f*_2_, the focal length of a lens. (d) Flowchart of the IBIFS method.

The schematic layout and optimization flow are depicted in Figure 1. The phase loaded on the SLM is a superimposition of a computer-generated hologram (CGH) [32] phase Φ_*I*_ and a Fresnel lens phase Φ_*F*_. The CGH phase, obtained from the test phase *φ*_*t*_ and the correction phase *φ*_*c*_, serves to separate the excitation beam into two paths: the test beam and the correction beam. The test beam is modulated with a series of preconfigured probing modes [21], [22], designed to interfere coaxially with the correction beam, aiding the gradual convergence of the correction beam to a focal point, as shown in Figure 1(b). The Fresnel lens phase allows the SLM to manually tune the focal position.

Figure 1(d) presents the IBIFS flowchart, illustrating the sequence of its operational steps. The correction phase is initialized to zero. The test beam is then loaded sequentially with different modes. During continuous image acquisition, image-based metrics are applied to evaluate the performance of the current test mode. The correction phase is recalculated iteratively using phase shifting method [33], [34].

(1) $$I_{p}={\left|{\tilde{T}}_{p}+\tilde{C}\right|}^{2}=t^{2}+c^{2}+{{\tilde{T}}_{p}}^{*}\tilde{C}+{\tilde{T}}_{p}{\tilde{C}}^{*}$$

Eq. (1) is the expression for a wide-field hologram formed by interference between the test beam with phase shifts and the correction beam. *I*_*p*_ is the intensity distribution of the hologram. The complex amplitude of the test beam with various phase shifts is represented as ${\tilde{T}}_{p}=t{\text{e}}^{\text{i}\left(\varphi_{t}+2\pi p/N\right)}$ , characterized by its amplitude *t* and phase distribution *φ*_*t*_. The phase shift step is 2π*p*/*N*, where *p* is a positive integer (*p* = 1, 2, …, *N*) representing the ordinal number of the phase shifts, and *N* is the number of phase steps. The theoretical minimum value of *N* is 3. However, we found through testing that *N* = 5 represents an optimal balance between measurement accuracy and measurement time. The complex amplitude of the correction beam is described as $\tilde{C}=c{\text{e}}^{\text{i}\varphi_{c}}$ with its amplitude *c* and phase distribution *φ*_*c*_.

By calculating the formula $1/N\sum_{p=1}^{N}{\tilde{T}}_{p}I_{p}$ , it can be found that this expression is numerically equivalent to the correction beam modulated by *t* ^2^:

(2) $$\frac{1}{N}\overset{N}{\underset{p=1}{\sum }}{\tilde{T}}_{p}I_{p}=t^{2}\tilde{C}$$

Eq. (2) indicates that the correction beam can be obtained from the preset test beam and the wide-field holograms in Eq. (1). However, wide-field holograms cannot be directly captured in a typical scanning TPM system, where the test beam is modulated by a wavefront shaper, and the emission signal is captured on the focal plane. The light field information at the conjugate plane of the SLM can only be inferred indirectly from the image quality at the focal plane. Therefore, an iterative algorithm is necessary for phase optimization based on image quality, which can be expressed as the following equation:

(3) $$\varphi_{c}=\arg\left({\tilde{C}}_{m}\right)=\arg\left({\tilde{C}}_{m-1}+\frac{1}{N}\overset{N}{\underset{p=1}{\sum }}{\tilde{T}}_{m,p}M_{m,p}\right)$$

In this context, *M*_*m*,*p*_ represents an image-based metric at the *m*th test mode and the *p*th phase shift. It should be noted that *I*_*P*_ in Eq. (2) signifies an array of the wide-field hologram distribution, while *M*_*m*,*p*_ in Eq. (3) corresponds to a single scalar value. A set of test modes is utilized to sequentially interfere with the correction beam. The correction phase is then refined after the completion of phase shift measurements for each mode. The interference of the two beams is achieved by the CGH, where the CGH phase pattern Φ_*I*_ loaded onto the SLM is given by the following equation [21]:

(4) $$\Phi_{I}=\arg\left[\sqrt{1-r}{\text{e}}^{\text{i}\varphi_{c,m}}+\sqrt{r}{\text{e}}^{\text{i}\left(\varphi_{t,m}+2\pi p/N\right)}\right]$$

In this equation, *m* denotes the ordinal number of the mode, and *r* is a constant between 0 and 1, used to adjust the weight of the two beams. To ensure convergence, the correction beam should be predominant, implying that *r* must be less than 0.5. Typically, *r* is set to a fixed value, depending on the test modes and the sample structure, with 0.3 being a common empirical value [21].

**Figure 2:**
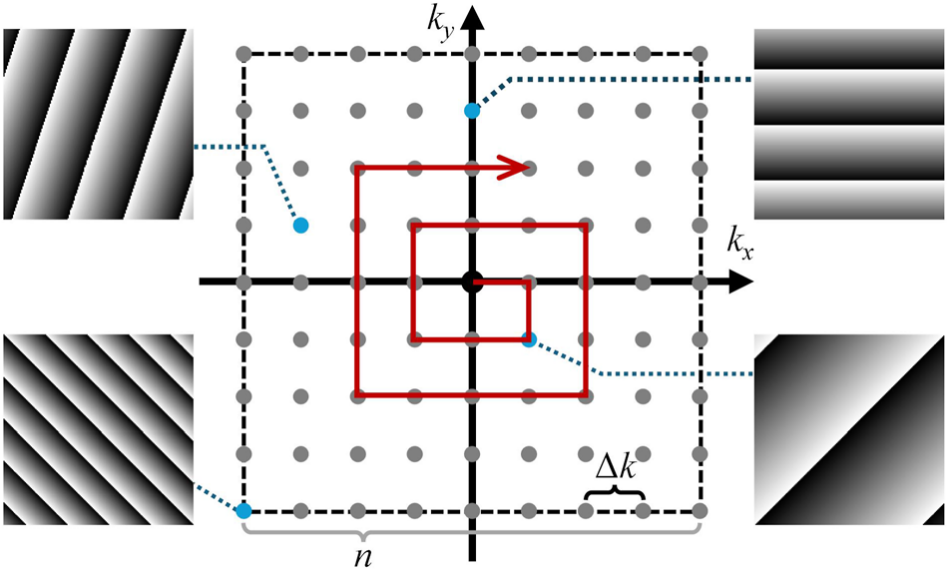
Schematic of the test modes. *n*, mode grid size, Δ*k*, mode interval.

The test mode is defined by the equation *φ*_*t*,*m*_ = *k*_*x*,*m*_ *x* + *k*_*y*,*m*_ *y*, i.e., tip-tilt wavefront, where *k*_*x*,*m*_ and *k*_*y*,*m*_ denote the spatial gradients in the *x* and *y* directions, respectively. The varying slopes mean that the test beam interferes with the correction beam at different offset angles. It is essential to select appropriate mode parameters according to the aberrations, such as the number and range of *k*_*x*_ and *k*_*y*_. It is worth noting that the discretization and order of the test modes influences the performance of the IBIFS. Figure 2 illustrates the schematic of the test modes, and the red arrow indicates the direction for sequentially loading the modes from the low spatial frequency components to the high spatial frequency components. Here, we define the mode interval Δ*k* = *k*_0_ *d*/*f*_*obj*_, where *k*_0_ is the vacuum wavevectors of light, *d* is a characteristic spatial sampling interval in the focal plane, and *f*_*obj*_ is the focal length of the objective lens. The total number of *n* ^2^ modes should cover an area in the focal plane approximately larger than the scattered PSF. The overall correction procedure is iterated at least twice to ensure convergence.

Four commonly used full FoV image-based metrics are employed in this paper as follows. SUM represents the aggregate intensity values across the entire FoV. This metric is considered more favorable for two-photon fluorescence but may be prone to shot noise for sparse samples. STD indicates standard deviation, a statistical metric commonly associated with image brightness that quantifies the degree of dispersion in an image. MHF indicates the middle-high spatial frequency components [35]. Compared to the low-frequency base and high-frequency noise, the medium frequency better reflects the characteristics of the image, and the choice of frequency range depends on the structure of the sample. PCA represents two-dimensional principal component analysis [36], which is widely used for dimensionality reduction. It can extract image features and serves as a metric correlated with spatial frequency.

To compare the IBIFS method with the DASH method, we employed a ROI-based metric as an approximation of DASH. In DASH, the system remains stationary at a single location, and the correction phase is iteratively updated based on the signal intensity at that location. Conversely, our system uses a resonant scanner that cannot be statically parked at different locations distributed over the entire FoV. So, the pixel dwell time (∼85 ns) is much shorter than in DASH (∼2 μs), resulting in a lower signal-to-noise ratio (SNR). Therefore, a small ROI (5 × 5 pixels, ∼2.3 × 2.3 μm^2^) with higher signal selected from a full FoV image (512 × 512 pixels, ∼255 × 255 μm^2^) is used for correction, with its mean intensity within the ROI used as the metric. In addition, the framerate of resonant-galvo scanners is at least one order of magnitude higher than that of galvo-galvo scanners. This is an advantage for the image-based AO configuration. The details of the system setup are presented in the next section.

## 3 Experimental setup

**Figure 3:**
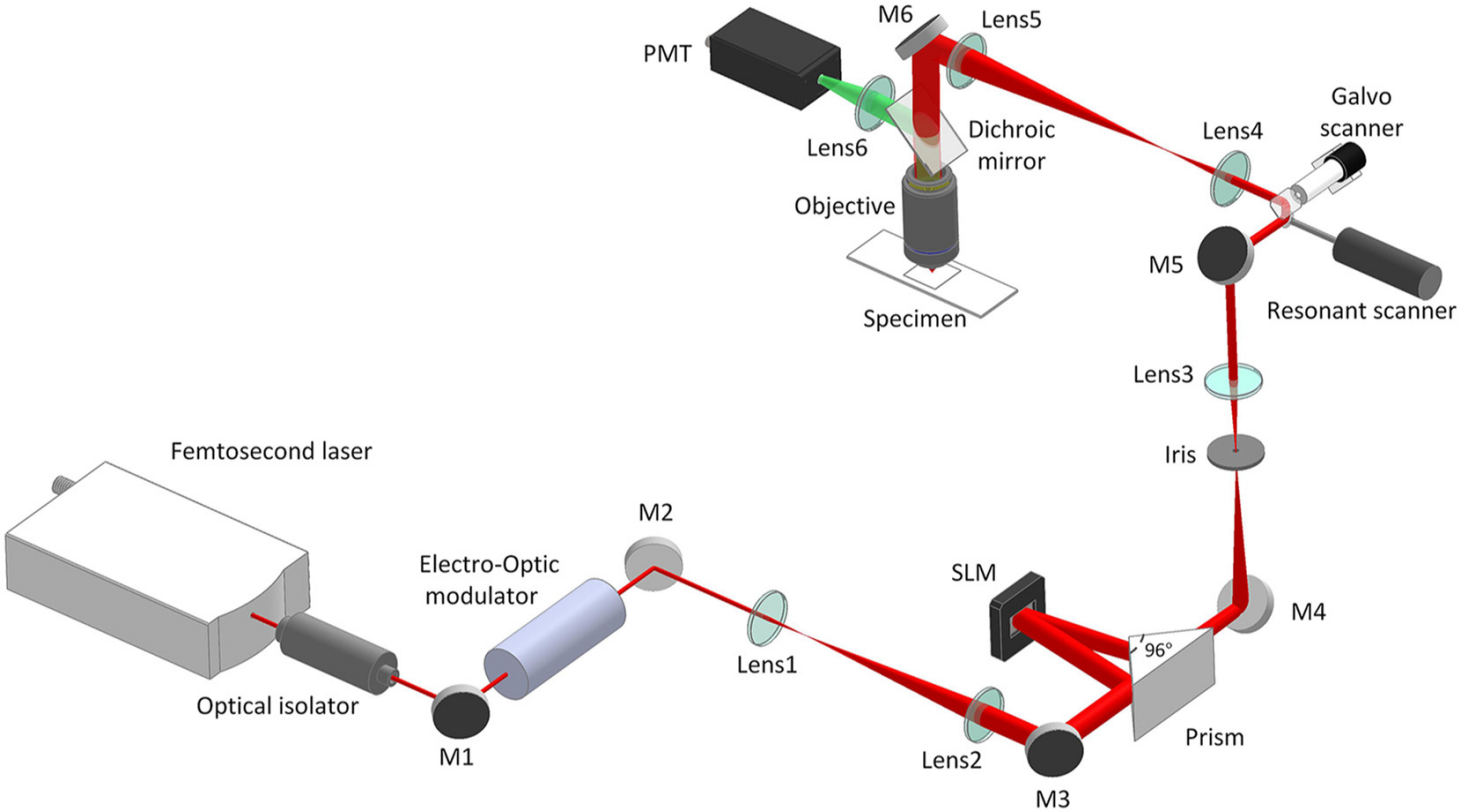
System configuration of the two-photon microscope with IBIFS method. M1–M6, mirrors; SLM, spatial light modular; PMT, photomultiplier tube.

A schematic diagram of the TPM imaging system with the IBIFS method is shown in Figure 3. A femtosecond laser (MaiTai HP, Spectra-Physics, USA) was used as the excitation light, and the wavelength was tuned to 780 nm for fluorescent beads imaging and 900 nm for mouse brain slice imaging. A Faraday isolator was employed to prevent back reflections, and an electro-optic modulator (M350-80, ConOptics, USA) was used for laser power modulation. The beam was expanded after passing through a 4*f* system consisting of two lenses (achromat doublet Lens1, *f* = 18 mm, achromat doublet Lens2, *f* = 100 mm). After passing through a polarizer, the beam is reflected by a 96° prism before reaching a liquid crystal spatial light modulator (X13139-07, Hamamatsu, Japan) from a slight angle and then reflected by the prism, which makes the optical layout compact. The phase patterns were updated at 5 Hz, which was limited by the slow response time of the SLM. The loaded phase pattern on the SLM consists of four main components: first, the phase of the blazed grating, used with an iris to remove the zero-order diffraction; second, the CGH phase for the optimization process; third, an optional artificial aberration phase for validation; fourth, a Fresnel lens (*f* = 800 mm) phase was loaded to form a converging wavefront and also to demagnify the beam with another lens (achromat doublet Lens3, *f* = 300 mm), to match the incident aperture of the resonant-galvo scanner (LSKGR12, 45 fps@512 × 512 pixels, Thorlabs, USA). The scanning plane was conjugated to the back aperture of the microscope objective by a scan lens (achromat doublet Lens4, *f* = 60 mm) and a tube lens (achromat doublet Lens5, *f* = 300 mm). A high numerical aperture (NA) objective lens (CFI Apochromat NIR 40 × W, 40×, Water Immersion, NA = 0.8, Nikon, Japan) was used for both the excitation of the sample and the collection of the fluorescence. The sample was mounted on a motorized XYZ stage (MP-285, Sutter Instrument, USA). The emission fluorescence was collected by a photomultiplier tube (H7422-50, Hamamatsu, Japan) after reflection by a dichroic mirror, where a lens (achromat doublet Lens6, *f* = 60 mm) was used to maximize the collection efficiency. Fluorescent spheres (FluoSpheres™ carboxylate fluorescent microspheres, 2.0 μm and 1.0 μm mixed, yellow-green, 505/515, Thermo Fisher Scientific, USA) embedded in agar were used in experiments for validation of our imaging system and methodology. All computations and hardware control were performed on a high-performance workstation (Dell Precision T7820, Intel Xeon Gold 2.3 GHz CPU with 64 GB memory, NVIDIA Quadro P4000 GPU).

## 4 Results

### 4.1 Effective FoV of IBIFS

**Figure 4:**
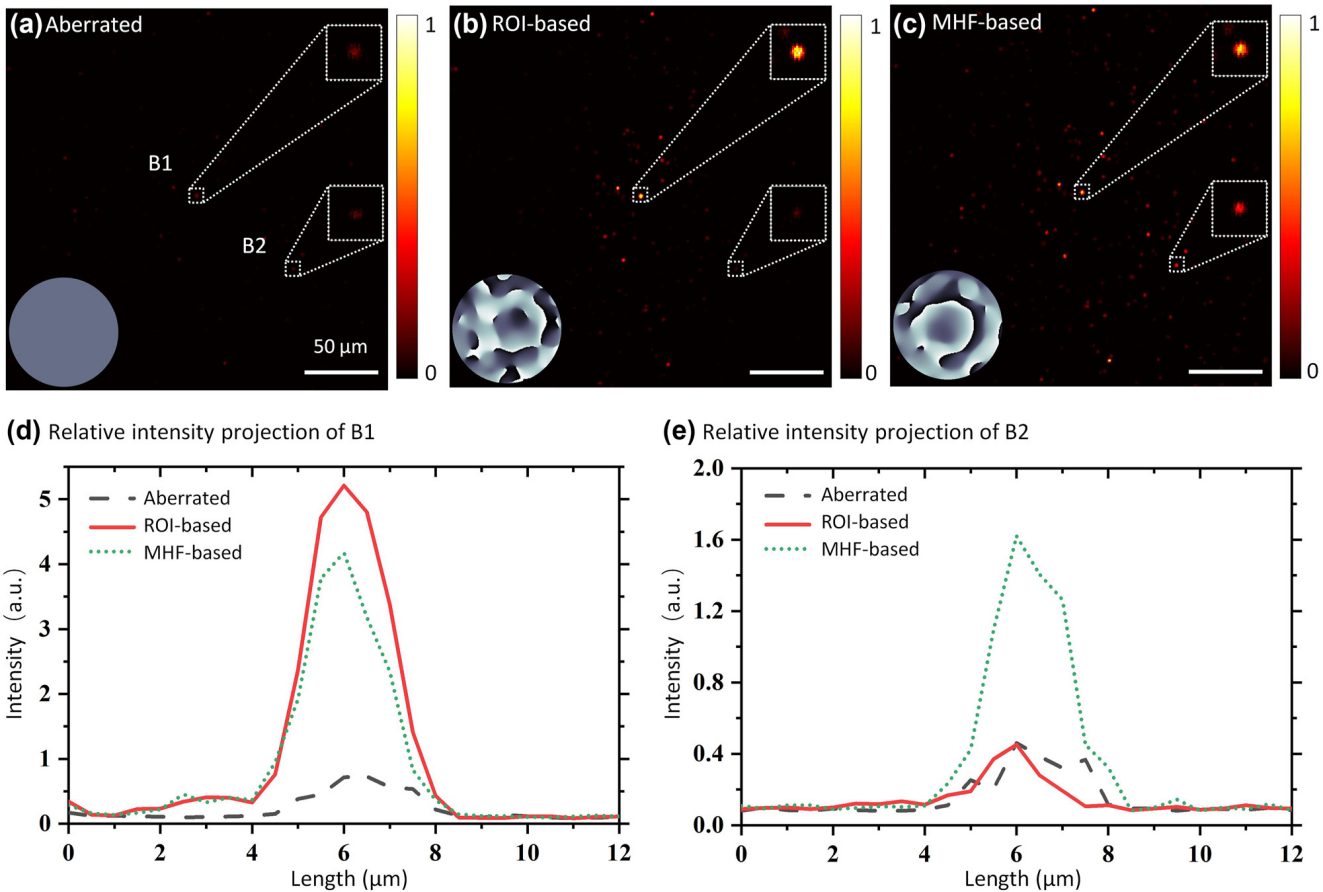
Experimental results of imaging fluorescent beads through a diffuser, corrected by the IBIFS with different metrics. Insets in (a)–(c) are the correction phases. (a) Image of fluorescent beads scattered by a diffuser. B1 and B2 are two fluorescent beads selected for comparison. (b) Image of fluorescent beads with correction based on the intensity value of B1. (c) Image of fluorescent beads with correction based on the middle-high spatial frequency characteristics. (d) Relative intensity profiles of B1. (e) Relative intensity profiles projection of B2.

We evaluated the impact of the metrics on the effective FoV through an aberration compensation experiment utilizing fluorescent beads. The number of modes was set to 100, and the correction process was repeated 3 times. The aberration was introduced by a holographic diffuser (1° diffusing angle, Edmund Optics, USA) placed above the sample. Figure 4(a) shows the image of the scattered fluorescent beads. Beads B1 and B2 serve as a comparison. B1, located in the central region of the fluorescent image, is regarded as a guide-star, with its mean intensity used as the ROI-based metric. B2 is situated in a peripheral region of the image. Figure 4(b) shows the corrected image based on the intensity value of bead B1. Figure 4(c) displays the correction results based on an image-based metric, with the correction phases shown in the respective insets. Comparing Figure 4(a) to (c), it is evident that only the area surrounding B1 is corrected in the ROI-based correction image, while the image-based method corrects the entire FoV. This distinction is further illustrated by comparing the two fluorescent beads B1 and B2. As shown in Figure 4(d), for the ROI-based method, the peak intensity of B1 is increased approximately 10-fold compared to the aberration state, whereas for the image-based method, the peak intensity of B1 is increased approximately 8-fold. The ROI-based method appears better at some local points. As a comparison, Figure 4(e) shows that the peak intensity of B2 in the image-based method is enhanced about 4-fold, while the ROI-based method shows no enhancement. The ROI-based method significantly improves the image quality for guide-star B1, while there is minimal correction for B2, which is distant from the guide-star. In comparison, the image-based method effectively improves the image quality for both B1 and B2. By using image-based metrics and conjugate AO configuration, the IBIFS offers a large effective FoV and may be beneficial for some applications.

In this experiment, approximately 5 min was required to optimize the correction phase with 3 iterations, 100 modes, and 5 phase shifts, and the refresh rate of the SLM was 5 Hz (total 3 × 100 × 5 × 0.2 s = 300 s). The overall correction time can be reduced by using a faster SLM.

### 4.2 Experiments of mouse brain slices with different metrics

**Figure 5:**
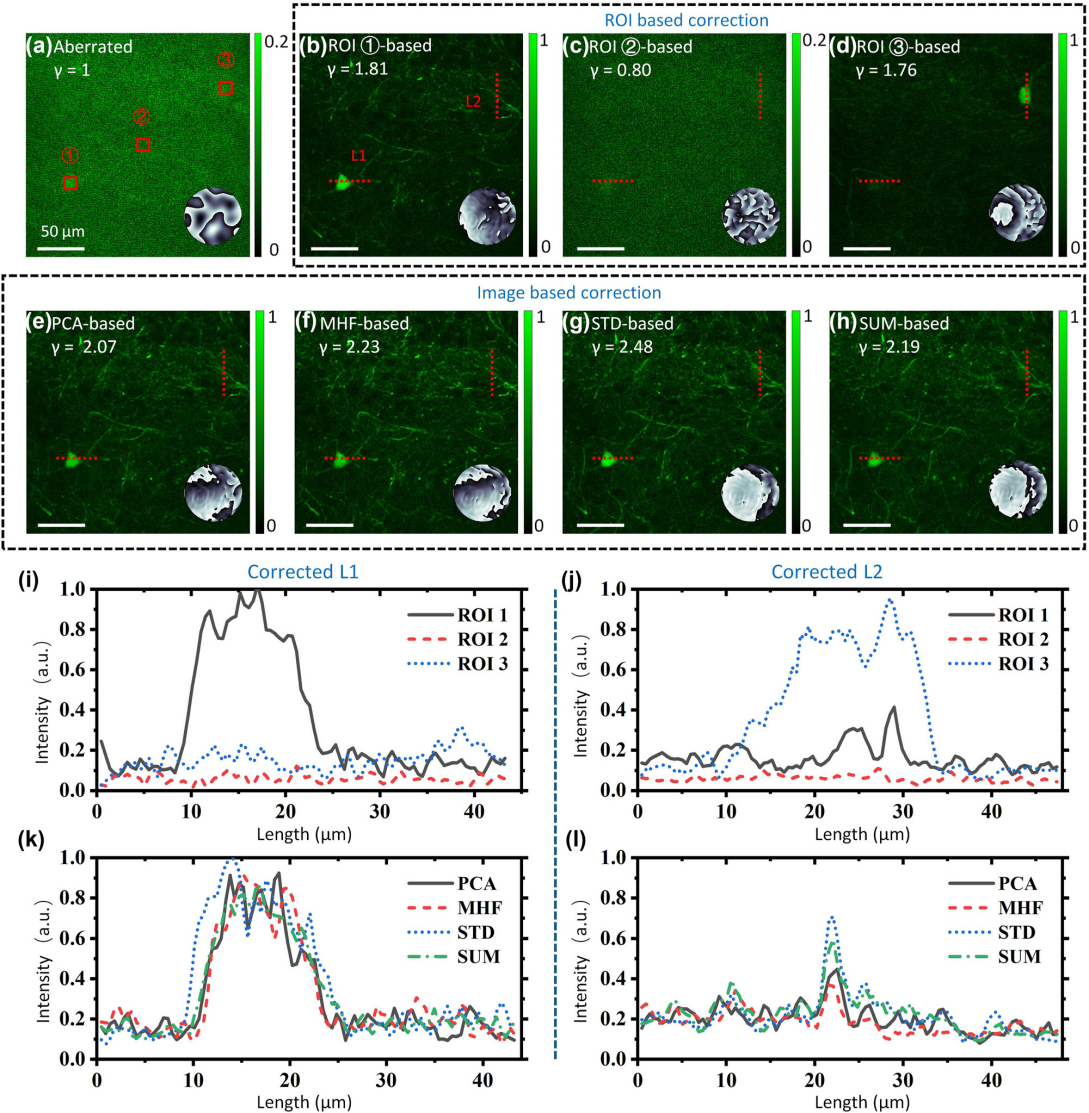
Scattering correction in mouse brain slice. (a) Aberrated image of the mouse brain. Three ROIs are labeled by red boxes. (b–d) Wavefronts corrected images based on the three ROIs in (a), *γ*, enhancement factor. L1 and L2 in (b) are two cross-sectional profile lines. (e–h) Wavefronts corrected images based on different image-based metrics. The insets of (a–h) illustrate the residual phases, and the residual phase in (a) is the initial aberration without correction, *σ* = 2, *β* = 2. (i) Corrected L1 based on three ROIs. (j) Corrected L2 based on three ROIs. (k) Corrected L1 based on image-based metrics. (l) Corrected L2 based on image-based metrics.

We compared the performance of different metrics using a green fluorescent protein (GFP) labeled mouse brain slice, as shown in Figure 5, where the wavefront distortion was artificially generated by applying Gaussian random phases [22] to the SLM. The random phase is determined by two parameters: the standard deviation *σ* that controls the spatial frequency components, and the magnitude factor *β* that scales the random phase in the range [0, 2*β*π]. In our experiments, we used *σ* = 2 and *β* = 2 to generate phase masks that resemble strong distortions. In this experiment, 196 modes were used for correction, and 3 iterations were performed. The enhancement factor *γ* is defined as the ratio of the total intensity of a corrected image to that of an aberrated image, as shown in Figure 5(a). Using the ROI-based metric, 3 ROIs are selected (red boxes) in Figure 5(a), and their corrected images are shown in Figure 5(b)–(d), respectively. Additionally, two cross-sectional profile lines, L1 and L2, are selected for analysis, as labeled in Figure 5(b). ROI1 has a higher SNR, resulting in an acceptable correction with a smoother residual phase, as shown in Figure 5(b). In contrast, the correction of ROI2 with low SNR is barely noticeable, as illustrated in Figure 5(c), where the mean intensity decreases, and the residual phase appears chaotic. Figure 5(d) illustrates a scenario where ROI3 contains strong signals but is situated in a plane that is axially displaced from the plane of ROI1. Although the image is corrected with relatively weak signal enhancement, a noticeable defocus phase is present in the residual phase. This indicates that the ROI-based IFS method is significantly influenced by the position of the ROI. The image presented in Figure 5(a) is distorted, making it difficult to extract obvious structural features for selecting appropriate ROIs, whereas the image-based approach avoids this limitation. The image-based correction brings better enhancement consistently with different metrics, as presented in Figure 5(e)–(h). The ROI-based method achieves a maximum *γ* value of only 1.81. In contrast, the image-based method consistently yields *γ* values above 2, with an optimal result of 2.48. This suggests that the image-based method is less susceptible to time-varying noise than the ROI-based approach. Furthermore, the optimization can be achieved by selecting the most appropriate metric based on the sample’s specific structure. Moreover, the sample structures in the corrected images are similar and the residual phases are nearly flat, showing that their wavefront correction results are robust. Figure 5(i) and (j) shows the intensity profiles L1 and L2 through each ROI. Intensity profiles with image-based metrics are shown in Figure 5(k) and (l). It is clear that the best correction is achieved only within the ROI selected for optimization. A subsequent comparison of the results in Figure 5(k) and (l) shows that the profiles exhibit a general similarity, indicating that the IBIFS method yields more stable and holistic outcomes.

### 4.3 Comparison of the test modes with different spatial sampling intervals

Fluorescent beads imaging experiments were conducted to demonstrate the role of test modes. Gaussian random phases were applied to the SLM to induce artificial aberrations. We compared the optimization processes using various spatial sampling intervals ranging from 0.5 μm to 1.25 μm with the same 10 × 10 sampling grids, totaling 100 modes. The optimization was repeated twice, resulting in a total of 200 measurements. The MHF metric served as the feedback. Each set of experimental conditions was repeated four times, and the total image intensity was recorded for each measurement.

**Figure 6:**
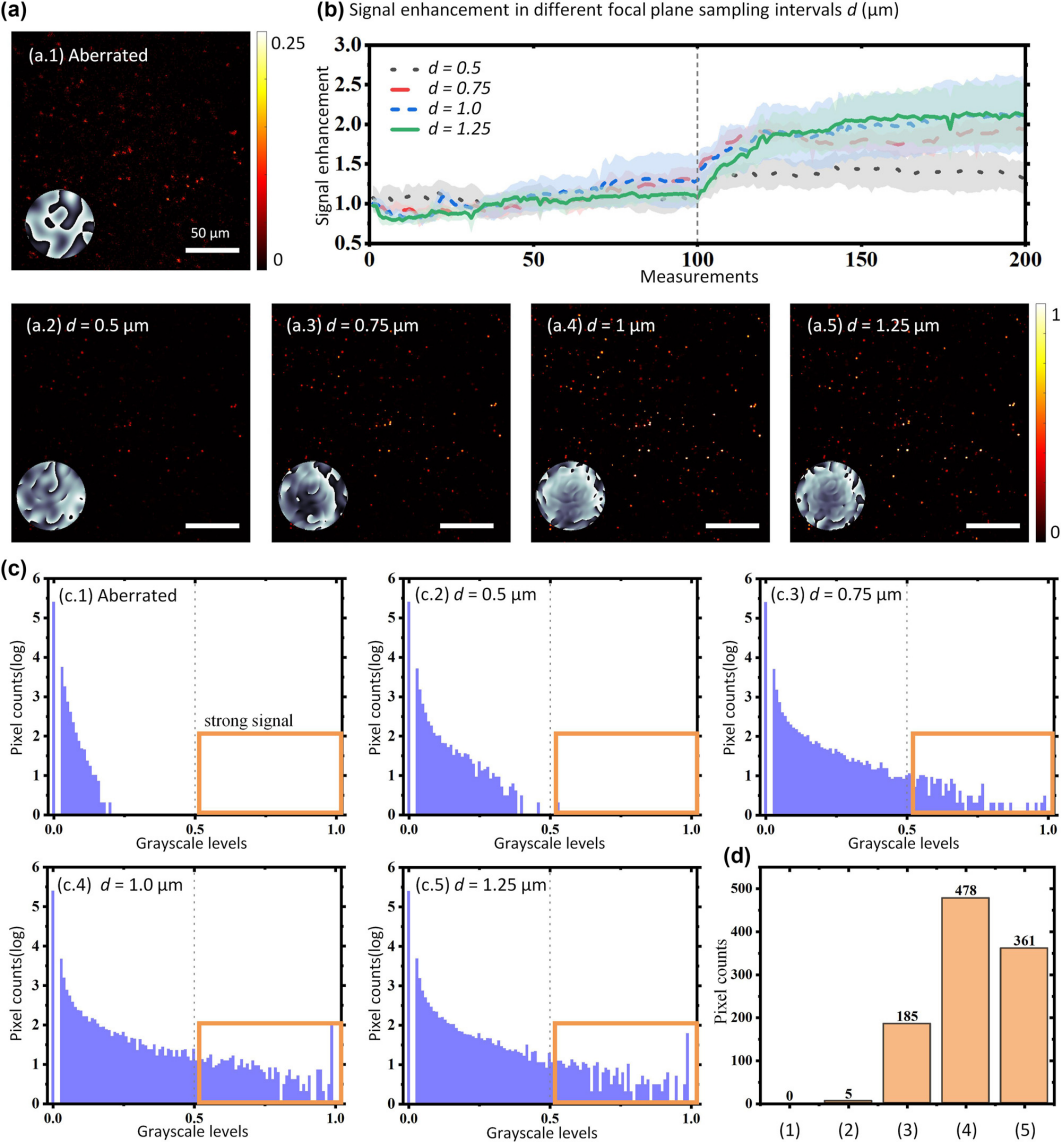
Comparison of the test modes with different spatial sampling intervals. (a) Normalized images of fluorescent beads, (a.1) aberrated image of mixed beads (2 μm and 4 μm), inset shows the applied artificial aberration phase on SLM, *σ* = 2, *β* = 2. (a.2–a.5) Wavefronts corrected images with different mode interval parameters, and insets showed the residual phase after correction. (b) Signal enhancement with different focal plane spatial sampling intervals. (c) Grayscale histograms of the images in (a), pixels with grayscale values exceeding 0.5 are identified as strong signal points, and a comparison of their counts is depicted in (d).

The scattered fluorescent beads are illustrated in Figure 6(a.1), accompanied by an inset that details the aberration phase. Figure 6(a.2) to 6(a.5) illustrates the corrected results for varying focal plane sampling intervals, each with an inset showing the corresponding corrected residual phase. The enhancement curves, which reflect the change in total image intensity across measurements under different sampling intervals, are presented in Figure 6(b). Additionally, to analyze the pixel intensity distributions of the images in Figure 6(a), their respective grayscale histograms are shown in Figure 6(c). Pixels with grayscale values above 0.5 are selected for comparison and are displayed in Figure 6(d). Examination of Figure 6(b) reveals that the enhancement curves stabilize at a higher level for sampling intervals of 1 μm and 1.25 μm. Furthermore, considering the strong signal points, Figure 6(d) indicates that the interval of 1 μm yields the highest count. It can be concluded that the sampling intervals of the test mode significantly influence the correction outcomes in IBIFS. For small sampling intervals, higher-order aberrations might not be sufficiently compensated, and significant high-frequency components remain in the residual phases depicted in Figure 6(a.2) to (a.5). On the contrary, if the interval is too large, some frequency component of the aberration might be missed. Adjusting the sampling interval while maintaining a constant number of modes is a convenient approach to achieve the optimal aberration correction effect. To address the challenge posed by deep tissue scattering, increasing the number of modes and iterations can further enhance the compensation effect. However, this also leads to an increase in the total correction time. Additionally, employing nonuniform mode sampling intervals or exploring alternative modes may offer further benefits, although these approaches warrant further investigation.

## 5 Discussion and conclusion

In this study, we proposed the IBIFS approach, which progressively corrects the wavefront over the entire FoV by monitoring the image-based metrics. Our approach was validated using fluorescent beads and mouse brain tissue. We quantified and compared the enhancement factors with several commonly used image-based metrics. The performance of the image-based and ROI-based methods was compared and analyzed, and the effect of test modes with different spatial sampling intervals on the correction effect was investigated.

DASH is exploited to provide a nonlinear feedback signal from the focal plane for the correction loop, which is different from regular modal decomposition in the pupil plane. The IBIFS approach is particularly suitable for resonant scanners due to their scanning characteristics. By using a resonant scanner, image-based metrics are readily accessible as feedback signals for wavefront correction. However, the resonant scanner cannot be arbitrarily parked at a specific location and the dwell time is far shorter than the requirement for successful optimization in DASH. Here, the image-based metrics of the acquired frames are fed to the DASH algorithm, thereby enabling the progressive enhancement of the full FoV image during the optimization process. A large correction area can be obtained by combining the conjugate AO setup and the image-based metrics. In addition, the full FoV enhancement factor of IBIFS is 37 % higher than that of the ROI-based DASH approach in experiments. The IBIFS correction procedure is also more stable and robust, producing consistent correction results.

In the common modal decomposition methods based on Zernike polynomials, the terms tip, tilt, and defocus are typically removed. These are called displacement modes or geometrical modes because they do not directly affect image quality, but cause a volumetric displacement. Even after the exclusion of these displacement modes, image displacement often still remains, with defocus having a particularly significant impact on microscopic imaging. In image-based AO methods, similar situations are more or less inevitable. As demonstrated in Section 4.2 of this paper, compared to IFS, IBIFS exhibits a stronger resistance to defocus distortion, but it is still affected by tip and tilt in practice. Furthermore, IBIFS can also be combined with other methods to further overcome this issue, such as the axially locked method mentioned in reference [37].

In conclusion, the IBIFS method has the ability to perform aberration correction for TPM, with the potential to enhance imaging capabilities in biological and other complex samples.
